# Mudstone creep experiment and nonlinear damage model study under cyclic disturbance load

**DOI:** 10.1038/s41598-020-66245-w

**Published:** 2020-06-09

**Authors:** Jun-guang Wang, Qing-lin Sun, Bing Liang, Peng-jin Yang, Qing-rong Yu

**Affiliations:** School of Mechanics & Engineering, Liaoning Technical University, Fuxin, Liaoning China 123000

**Keywords:** Solid Earth sciences, Materials science

## Abstract

To study the creep characteristics of mudstone under disturbed load, creep rock triaxial compression disturbance tests under different disturbance amplitudes and frequencies are conducted using a self-made triaxial disturbed creep test bench for rock. The influence of different factors on the creep deformation law of each stage is analyzed. The results show that the disturbance effect has a significant impact on the creep properties of mudstone, and various factors have different effects on the creep stages. The instantaneous deformation variable, creep decay time, and steady creep rate change exponentially with the increase in axial pressure, and increase linearly with the increase in disturbance amplitude and disturbance frequency. The disturbance amplitude has a more significant effect on the instantaneous deformation, steady-state creep rate, and accelerated creep. According to the analysis of the test results, a nonlinear disturbance creep damage model based on Burger’s model is established. The model is identified and calculated by the improved least squares method based on pattern search. The influence of different disturbance factors on the creep parameters is analyzed. The model fitting results and experimental results are compared to demonstrate that the model is used to simulate different disturbances. It was observed that rock creep under certain conditions exhibits certain adaptability. It is of great significance to carry out rock disturbance creep experiments and study the theory of disturbance creep to ensure the long-term stability of deep rock mass in complex environment.

## Introduction

With the gradual depletion of shallow resources and development of resources, the number of coal mines with a depth of more than one kilometer is increasing in China. Owing to the particularity of the environment and the complexity of the stress field of deep rock mass, specifically when the deep rock mass is in a complicated environment, the stress state of some middle strength roadways that surround rock has approached the limit of coal and rock strength; thus, these roadways are vulnerable to instability and failure caused by tunneling or mining disturbances^1^. Creep refers to the phenomenon of accumulation of permanent strain with time under a constant external force. The creep characteristics of rock are one of the important properties of rock mechanics. Therefore, studying the creep mechanical properties of rock under load disturbances has important practical value for ensuring the long-term stability of rock mass.

Numerous scholars have conducted extensive research in the abovementioned fields and obtained useful results. Jindřich Šancer and Bagde M.N.^2–4^ qualitatively studied and analyzed the rheological behavior of sandstone under cyclic loading with variable amplitudes and frequencies. The results showed that the creep properties of sandstone depend strongly on the stress state of rock. Gao Yangfa and Wang Bo^5,6^ developed an instrument based on rheological disturbance and used it to test rock rheology and its disturbance effect. The disturbance effect of rock rheology was proposed, and the factors that influenced this effect were analyzed. Cui Xihai^7^ conducted an impact load creep test on mudstone and established a disturbance creep rheological model. Song Dazhao^8^ studied the creep damage characteristics of a coal-bearing rock mass under disturbing actions. Pu Chengzhi and Wang Qihu^9,10^ considered the process of rock damage and cracking and established nonlinear creep damage models respectively.

Throughout the above studies, most scholars examined the disturbance load when the disturbance creep is applied by an impact load and rarely analyzed the rock disturbance creep laws with respect to disturbance amplitude and the disturbance frequency. Therefore, in this study, based on the previous research, a mudstone disturbance creep test was conducted under different axial pressures, disturbance amplitudes, and disturbance frequencies. The influence of various factors on the creep characteristics was analyzed, and on this basis, a nonlinear perturbation creep damage model was established. The ultimate objective of this study was to provide basic data for studying the creep mechanical properties of deep rock masses in complex environments.

## Methods

### Mudstone disturbance creep test

#### Disturbance load and Disturbance creep

**Figure 1:**
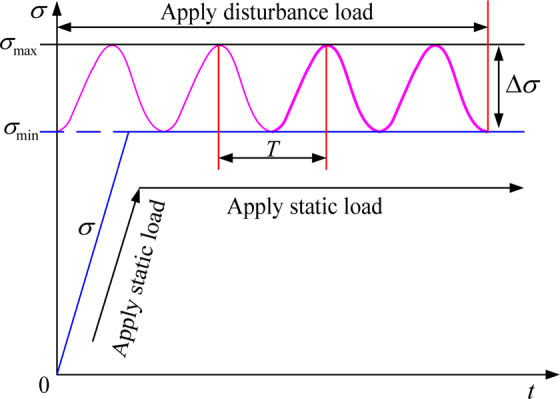
Schematic diagram of load application.

Disturbance load refers to a group or groups of loads acting on rock only in an instant or within a certain time interval. Passing this instant or time interval, the group or groups of loads disappear immediately. Disturbance creep refers to the deformation of rock under a certain stable stress state during the action of a certain disturbance load. The disturbance load in this paper is a sinusoidal disturbance load with different disturbance amplitude and frequency applied to mudstone under certain axial pressure and confining pressure. The loading process is shown in Fig. 1.

#### Mudstone disturbance creep test equipment

**Figure 2:**
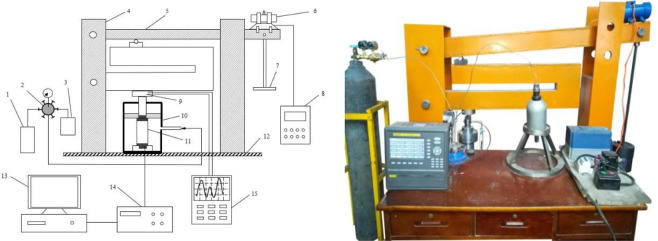
Disturbance creep test rig. (1) Air pump. (2) Six-way valve. (3) Stabilizer tank. (4) Pillar. (5) Rigid beam. (6) Vibrator. (7) weight bench. (8) Frequency modulator. (9) GH-4 pressure sensor. (10) Triaxial pressure chamber. (11) Rock specimen. (12) Base. (13) Computer. (14) Dynamic strain gauge. (15) TOPRIE multiplex data recorder.

The test equipment is a rock triaxial disturbance creep test rig, which comprises a triaxial pressure chamber, an axial compression and confining pressure loading system, a disturbance loading and a data monitoring system. The test equipment is shown in Fig. 2. The axial compression loading is realized by adding weights on the weight bench, and the weights conduct the pressure to the triaxial pressure chamber through the lever structure, thus attaining the purpose of applying axle load to the test piece. The confining pressure is loaded by injecting nitrogen through an air pump, and the range of confining pressure loading is 0~10 MPa; The disturbance loading system is composed of YZU-30-6B vibration motor and SRMCO-VM05 frequency converter. The disturbance amplitude is adjusted by adjusting the angle of the rotor of the vibration motor. The disturbance frequency is adjusted by adjusting the SRMCO-VM05 frequency converter, the disturbance frequency range is 0~20 Hz; The data monitoring system consists of TOPRIE multiplex data recorder and DHDAS dynamic strain gauge. The top of the triaxial pressure chamber is equipped with GH-4 pressure sensor, and GH-4 pressure sensor is connected with TOPRIE multiplex data recorder. The real-time axial pressure data of the sample can be viewed on the TOPRIE multiplex data recorder. The accuracy of axial pressure measurement is not more than 0.05% of the measuring range and the minimum resolution is 0.6 N. The change signal of the sample strain is transmitted to DHDAS dynamic strain gauge through BX120-20AA strain gauge. The other end of DHDAS dynamic strain gauge is connected to a computer. The real-time strain data of rock samples can be viewed at the computer end. The strain measurement accuracy is not more than 0.5% ± *μm* of the measuring range and the minimum resolution is 0.1 *μm*.

#### Test scheme and process

The rock samples used in the test were cores of mudstone obtained from the Pingdingshan No. 12 mine, the sampling depth was 759 m, and the average density was 2.31 g/cm^3^. Mudstone is mainly composed of clastic minerals, clay minerals and carbonate minerals, and contains a small amount of pyrite, which is easy to expand when encountering water. The rock samples were ground to the specifications of the international standard (Φ: 50 mm × 100 mm). Discrete samples were eliminated after ultrasonic testing, and 12 samples with good consistency were selected for testing. Six specimens were selected for mudstone uniaxial and triaxial compression tests. The mudstone disturbance creep test used a grading loading method to conduct triaxial compression creep tests with different axial pressures, disturbance amplitudes, and disturbance frequencies. According to the basic mechanical properties of the mudstone, the axial pressure and confining pressure were initially set to 10 and 3 MPa, respectively. The confining pressure was maintained constant, and 4 leves of axial pressure were gradually added in loading gradients of 5 MPa. The disturbance amplitudes were 1.6, 3.2, and 4.8 MPa, and the disturbance frequencies were 1, 3, and 5 Hz. The mudstone disturbance creep test scheme is shown in Table 1.

**Table 1:** Scheme of disturbance creep test.

Influence factor	Sample number	Confining pressure (σ/MPa)	Disturbance amplitude (Δσ/MPa)	Disturbance frequency (ƒ/Hz)
Undisturbed	N1	3	—	—
Amplitude Frequency	N2	3	1.6	3
	N3	3	3.2	3
	N4	3	4.8	3
	N5	3	3.2	1
	N6	3	3.2	5

The specific test process is as follows:

The model BX120-20AA strain gauge is selected and pasted in the middle of the test piece and the relative position of the strain gauge is “T” shape. After the test piece is sealed by thermoplastic sleeve, it is placed into the triaxial pressure chamber, and the air tightness is checked. The TOPRIE multiplex data recorder and the DHDAS dynamic strain gauge are connected. The vibration motor is connected to the inverter, and the amplitude and frequency of the disturbance power are adjusted to the specified values. Simultaneously, the axial pressure and the confining pressure are applied and maintained constant after adding the required value. The disturbance load is applied, the next axial load is applied after 5 hours. The same specimen disturbance amplitude and frequency remain unchanged, and the confining pressure is maintained constant during the test; When the strain of rock increases with time, the rock will enter the stage of accelerated creep, until the rock has a one-time fracture surface, and the rock creep experiment ends; the stress and strain data are read and processed to analyze the creep characteristics of the mudstone by different factors. This experiment is carried out at room temperature, and the rock sample is in the state of natural water content.

## Results and Discussion

### Analysis of mudstone disturbance creep test results

#### Analysis of creep curve characteristics

**Figure 3:**
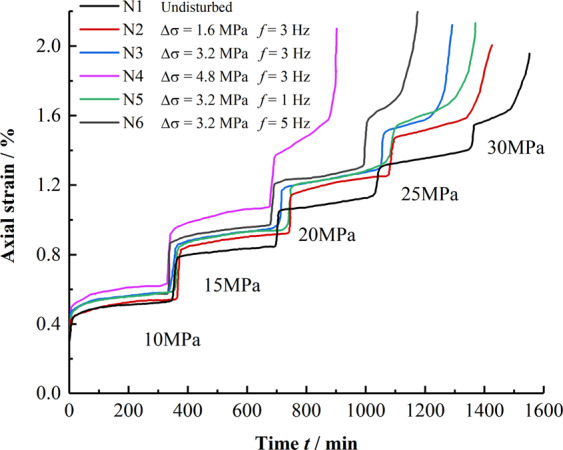
Creep curves under various conditions.

The creep curves of mudstone under different disturbance conditions according to the data of the axial strain *ε* and creep time *t* of mudstone under different disturbance conditions are plotted in Fig. 3.

1. When the axial load is applied, the samples produce obvious transient strain and then enter the creep attenuation phase. Under a lower axial stress, the creep rate of the mudstones tends to be stable, and as the axial pressure increases, the creep rate of mudstone increases, and the mudstone finally enters the accelerated creep stage, resulting in large deformation in a short time.
2. Creep deformation at a constant stress is smaller without perturbation than that with disturbance. Compared with the undisturbed condition, the time and stress required for mudstone to reach the same creep deformation under the disturbed condition are reduced.
3. For disturbance amplitudes of 1.6, 3.2, and 4.8 MPa, the critical axial stress at which the sample enters the accelerated creep stage are 25, 25, and 20 MPa, respectively, and the axial stress value is 25 MPa; when the disturbance frequencies are 1, 3, and 5 Hz, respectively, the rock enters the accelerated creep state. As can be seen, with the increase of disturbance amplitude, the stress threshold value of mudstone entering accelerated creep decreases while the disturbance frequency has no effect on the stress threshold value of mudstone entering accelerated creep.

#### Instantaneous deformation law

**Figure 4:**
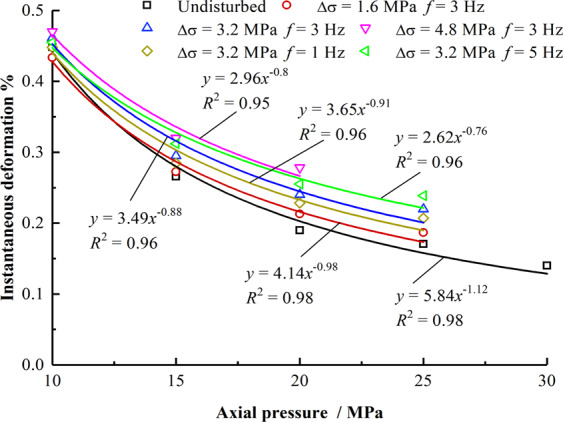
Instantaneous deformation variable with axial pressure.

Instantaneous deformation refers to the instantaneous deformation of rock at the initial stage of loading, which is mainly due to the deformation caused by the tight closure of the internal pores and fissures of the rock. Figure 4 indicates that under undisturbed and disturbed conditions, the instantaneous deformation of each mudstone specimen changes with a negative exponential function as the axial pressure increases. According to the fitting analysis, the relationship between the instantaneous deformation of mudstone, the axial stress and the frequency and amplitude of the stress perturbation is as follows:

1 $$\Delta \varepsilon ={p}_{1}(\Delta \sigma ,f){\sigma }_{1}^{-{q}_{1}(\Delta \sigma ,f)}$$

2 $${p}_{1}(\Delta \sigma ,f)=0.0901\Delta {\sigma }^{2}-0.0811{f}^{2}-1.03\Delta \sigma +0.218f+5.93,\,{R}^{2}=0.998$$

3 $${q}_{1}(\Delta \sigma ,f)=-0.00401\,\Delta {\sigma }^{2}-0.0112{f}^{2}+0.0829\Delta \sigma -0.0271f-\,1.13,\,{R}^{2}=0.99$$

In the equations, Δε is the instantaneous strain, Δσ is the disturbance amplitude, *f* is the disturbance frequency, ${p}_{1}(\Delta \sigma ,f)$ and ${q}_{1}(\Delta \sigma ,f)$ is a functional relationship with Δσ and ƒ, respectively. ${p}_{1}(\Delta \sigma ,f)$ and ${q}_{1}(\Delta \sigma ,f)$ are shown in Eqs. (2) and (3).

**Figure 5:**
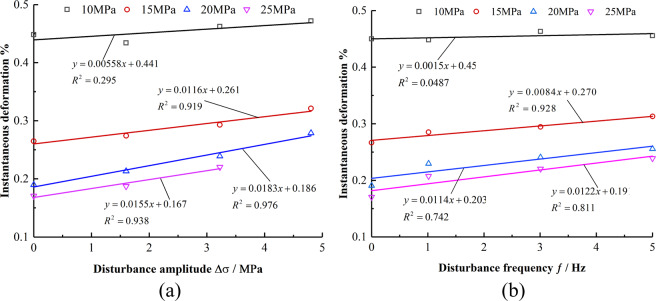
Relationship between instantaneous deformation and disturbance amplitude and frequency. (**a**) Relationship between instantaneous deformation variable and Δ*σ*. (**b**) Relationship between instantaneous deformation variable and *f*.

Figure 5(a,b) indicate that when the axial pressure is constant, the instantaneous deformation variable Δε of the mudstone has a positive correlation linear relationship with an increase in the disturbance amplitude Δσ and the disturbance frequency. When the axial pressure is 15, 20, and 25 MPa, the slopes of Δε with Δσ and ƒ increase are 0.0116, 0.0183, 0.0155 and 0.00840, 0.0114, 0.0122, respectively, which indicates that the instantaneous deformation of mudstone is more sensitive to an increase in the disturbance amplitude.

#### Rules of creep decay time

**Figure 6:**
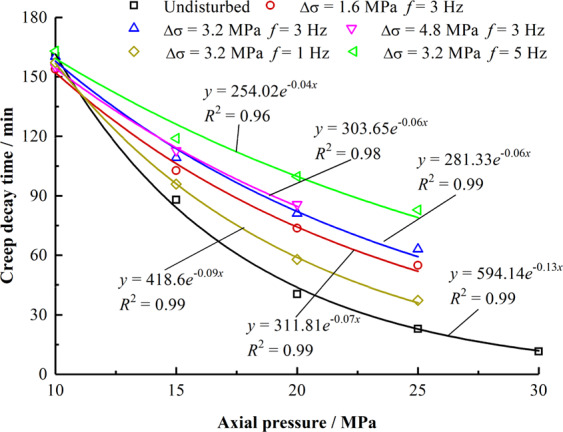
Relationship between creep decay time and axial pressure.

After instantaneous compaction of rock, mudstone enters the stage of decay creep, in which the creep rate of rock decreases with time. When the creep rate decreases to a steady state, the decay creep stage ends. The time is the decay creep time. The creep decay time decreases with a negative exponential relationship as the axial stress increases (as shown in Fig. 6) and tends to be stable. The relationship between the decay time of mudstone creep and the axial stress is as follows:

4 $${t}_{r}={p}_{2}(\Delta \sigma ,f){e}^{-{q}_{2}(\Delta \sigma ,f){\sigma }_{1}}$$

where *t*_*r*_ is the creep decay time, Δσ is the disturbance amplitude, ƒ is the disturbance frequency. ${p}_{2}(\Delta \sigma ,f)$ and ${q}_{2}(\Delta \sigma ,f)$ are functional relationships with Δσ and ƒ respectively. ${p}_{2}(\Delta \sigma ,f)$ and ${q}_{2}(\Delta \sigma ,f)$ are shown in Eq. (5) and Eq. (6).

5 $${p}_{2}(\Delta \sigma ,f)=363\Delta {\sigma }^{2}+11.1{f}^{2}-30.1\Delta \sigma -108f+585\,{R}^{2}=0.961$$

6 $${q}_{2}(\Delta \sigma ,f)=2.13\times {10}^{-4}\Delta {\sigma }^{2}+0.0018{f}^{2}-0.00286\,\Delta \sigma -0.0237f+0.129,\,{R}^{2}=0.932$$

**Figure 7:**
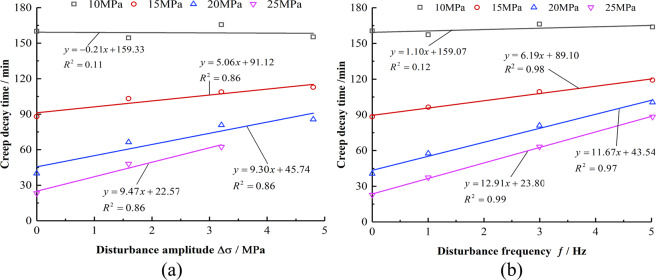
Relationship between creep decay time and disturbance amplitude and frequency. (**a**) Relationship between creep decay time and Δ*σ*. (**b**) Relationship between creep decay time and *f*.

Figure 7(a,b) show that when the mudstones experienced the same axial stress, the creep decay time shows a positive linear relationship with an increase in the disturbance amplitude and frequency. When the axial stress values are 15 MPa, 20 MPa, and 25 MPa, the slopes of the linear relationship of creep decay time with Δσ and ƒ are 5.06, 9.30, and 9.47 and 6.19, 11.67, and 12.91, respectively. When the stress state remains unchanged, the influence of the disturbance frequency on the creep decay time is more considerable than the disturbance amplitude.

#### Laws of steady state creep rate

**Figure 8:**
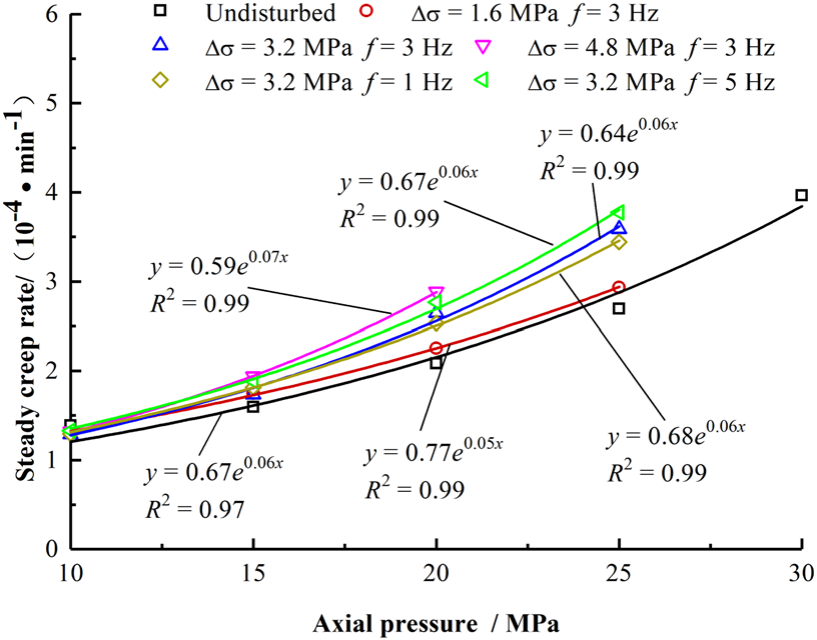
Relationship between steady-state creep rate and axial pressure.

After stage of the decay creep, the rock enters the steady creep stage, in which the rock creep rate is almost unchanged at the same stress level and the creep curve is straight. The steady creep rate rises with the increase of axial pressure. Figure 8 shows that under different disturbances, the steady state creep rate of mudstone increases exponentially with the increase in axial stress. The steady state creep rate can be obtained through the fitting analysis; further, the relationship between the axial pressure changes is as follows:

7 $$\mathop{\varepsilon }\limits^{\cdot }={p}_{3}(\Delta \sigma ,f){e}^{-{q}_{3}(\Delta \sigma ,f)\cdot {\sigma }_{1}}$$

8 $${p}_{3}=-0.0144\Delta {\sigma }^{2}+0.00333{f}^{2}+0.0581\Delta \sigma -0.0324f+0.680,\,{R}^{2}=0.996$$

9 $${q}_{3}=0.00172\Delta {\sigma }^{2}-3.53\times {10}^{-5}{f}^{2}-0.00418\Delta \sigma +0.00194f+0.0579,\,{R}^{2}=0.994$$

$\dot{\varepsilon }$ is the steady state creep rate, Δσ is the disturbance amplitude, *f* is the disturbance frequency. ${p}_{3}(\Delta \sigma ,f)$ and ${q}_{3}(\Delta \sigma ,f)$ are the functional relationships between Δσ and ƒ respectively. ${p}_{3}(\Delta \sigma ,f)$ and ${q}_{3}(\Delta \sigma ,f)$ are shown in Eqs. (8) and (9).

**Figure 9:**
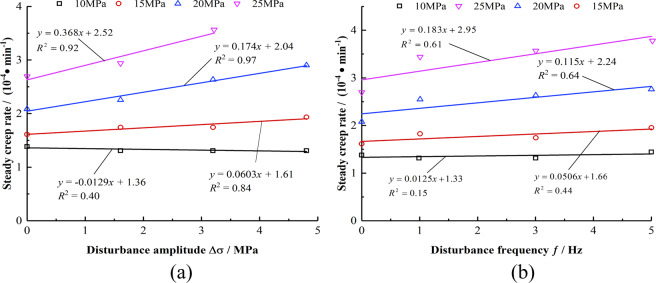
Relationship between steady creep rate and disturbance amplitude and frequency. (**a**) Relationship between steady-state creep rate and Δ*σ*. (**b**) Relationship between steady-state creep rate and *f*.

From Fig. 9(a,b), the steady creep rate increases linearly with the increase in the disturbance amplitude and frequency under the same axial stress. The fitting relationship in the figure shows that when the axial stress values are 15 MPa, 20 MPa, and 25 MPa, the slopes of the steady-state creep rate with the increase in Δσ and ƒ are 0.0603, 0.174, and 0.368, and 0.0506, 0.115, and 0.183, respectively. Therefore, the influence of the disturbance amplitude on the steady-state creep rate is more significant, and this effect becomes increasingly evident as the axial stress increases.

#### Laws of accelerated creep deformation

When the creep rate of rock increases sharply with time, the rock begins to enter the accelerated creep stage. With time, strength of the rock decreases. When the strength of the mudstone is lower than the applied load, the mudstone begins to fail. Under the disturbance load, the damage caused by different degrees of disturbance to the mudstone is also different, especially in the accelerated creep stage.

**Figure 10:**
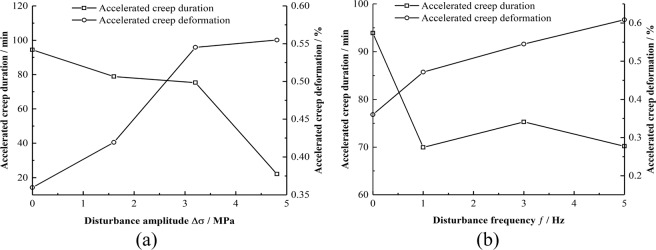
Accelerated creep law under different conditions. (**a**) Different disturbance amplitude. (**b**) Different disturbance frequencies.

When the disturbance amplitude values are 1.6, 3.2, and 4.8 MPa, the time periods at which the mudstone enters the accelerated creep phase are 1338 min, 1253 min, and 878 min, respectively. When the disturbance frequency is 1, 3, and 5 Hz, the time periods at which the mudstone enters the accelerated creep phase are 1294, 1253, and 1114 min, respectively. In the accelerated creep phase, the creep holding time and amount of deformation under different disturbance conditions are also different (see Fig. 10). The figure shows that the acceleration creep variable increases with disturbance amplitude and frequency. Figure 8(a) shows that as the disturbance amplitude increases, the acceleration creep time decreases. When Δσ = 4.8 MPa, the acceleration creep phase lasts only 21 min, with the disturbance. With an increase in frequency, changes of the accelerated creep phase of the mudstone is not evident, and the acceleration creep duration fluctuates around 70 min.

### Establishment of mudstone disturbing creep damage model

#### Proposed creep damage model

According to the analysis of mudstone creep disturbance test results under different disturbance conditions, the creep process of mudstone under the disturbing action has the following characteristics:

1. Part of the elastic strain is generated during transient loading, and elastic elements should be present in the model;
2. After loading, the mudstone deforms instantaneously and then enters the stage of decay creep. As time goes on, the creep rate of mudstone tends to be stable and the rock enters the steady creep stage, the model should contain viscous components;
3. With an increase in axial stress, the increase in strain with time does not tend to stabilize and the rock is finally fails. However, the rock has an irreversible accelerated creep with viscoplasticity;
4. Under different stress and disturbance conditions, the creep characteristics of mudstone change significantly, that is, the creep parameters of mudstone change with stress, time, and disturbance; thus, the model should include the damage factors considering the deterioration of rock parameters.

**Figure 11:**
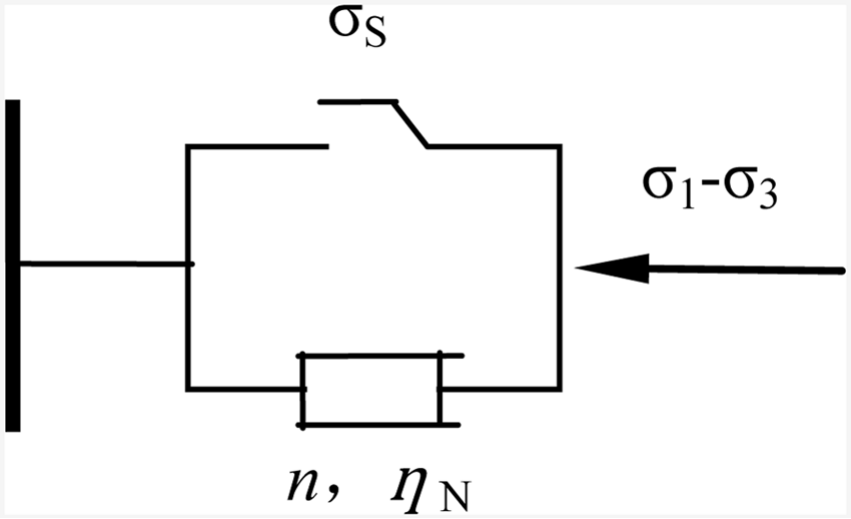
Nonlinear viscoplastic creep components.

Burger’s model can effectively reflect the decay and steady creep stages of rock creep process; however, it cannot describe the acceleration phase of creep. Therefore, the nonlinear viscoplastic creep element (NVPB body) is introduced according to the literature^11^, and this element is connected in series with Burger’s model; thus, the whole process of creep can be described more completely. A nonlinear viscoplastic creep element (NVPB body) is shown in Fig. 11.

The creep equation of the NVPB body under stress is as follows:

10 $$\varepsilon (t)=\{\begin{array}{cc}0 & {\sigma }_{0} < {\sigma }_{S}\\ \frac{{\sigma }_{0}-{\sigma }_{s}}{{\eta }_{N}}{(t-{t}_{{\rm{N}}})}^{n} & {\sigma }_{0}\ge {\sigma }_{S}\end{array}$$

In the equation, ${\sigma }_{0}={\sigma }_{1}-{\sigma }_{3}$, ${\sigma }_{1}$ is the first principal stress, ${\sigma }_{3}$ is the third principal stress; ${\sigma }_{S}$ is the stress threshold; *t* is the creep time; ${\eta }_{{\rm{N}}}$ is the NVPB body viscosity coefficient; and *n* is the creep index, which *t*_*N*_ is the entry point of the accelerated creep time.

The test results show that the creep characteristics of mudstone under the disturbance state are related to loading time, axial stress, disturbance amplitude, and disturbance frequency. To establish a mudstone creep damage model that can reflect transient, decay, steady, and accelerated creep, this study introduces damage variables according to Kachanov^12^ creep damage theory, in which the damage factor should be a function of axial stress, disturbance amplitude, disturbance frequency, and creep time, as described below:

11 $$D=f({\sigma }_{1},\Delta \sigma ,f,t)$$

**Figure 12:**
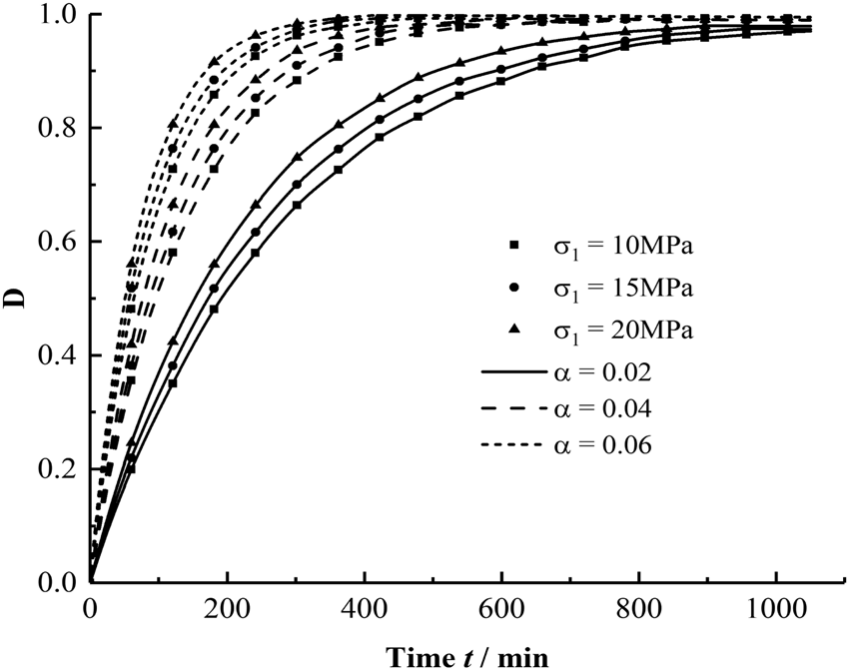
D curves with time for different *α* and *σ*_1_.

According to the research results of a large number of rock creep damage tests, the creep damage variable of rock increases with time in the negative exponential function during the creep process^13,14^. In this study, the damage variable is reduced to Eq. 12.

12 $$D=1-{{\sigma }_{1}}^{-\alpha (\varDelta \sigma ,f)t}$$

where *α* is a rock damage parameter affected by the disturbance frequency and amplitude. As the values of *α* and *σ*_1_ are different, the damage factor *D* varies with time *t*; the trend is shown in Fig. 12.

**Figure 13:**
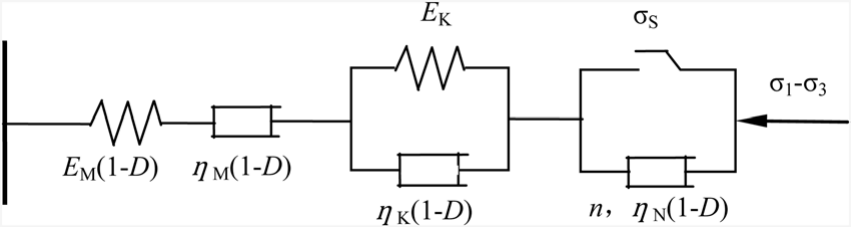
Improved disturbance-visco-elastoplastic damage creep model.

The nonlinear viscoelastic-plastic creep model is modified for the creep properties of mudstone under disturbance. The model consists of a modified Maxwell body, modified Kelvin, and modified nonlinear viscoplastic body. The schematic diagram of the model is shown in Fig. 13, where 1 and 2 are the elastic modulus and viscosity coefficient of the Maxwell body, and 3 and 4 are the elastic modulus and viscosity coefficient of the Maxwell body, respectively.

It can be concluded that the creep equations of the mudstone creep damage model are as follows:

(1) When there is no disturbance ($\Delta \sigma =0$, $f=0$):

13 $$\varepsilon (t)=\frac{{\sigma }_{1}-{\sigma }_{3}}{{E}_{{\rm{M}}}}+\frac{{\sigma }_{1}-{\sigma }_{3}}{{\eta }_{{\rm{M}}}}t+\frac{{\sigma }_{1}-{\sigma }_{3}}{{E}_{{\rm{K}}}}(1-{e}^{\frac{-{E}_{{\rm{K}}}}{{\eta }_{{\rm{K}}}}t})\,{\sigma }_{1}-{\sigma }_{3} < {\sigma }_{S}$$

14 $$\varepsilon (t)=\frac{{\sigma }_{1}-{\sigma }_{3}}{{E}_{{\rm{M}}}}+\frac{{\sigma }_{1}-{\sigma }_{3}}{{\eta }_{{\rm{M}}}}t+\frac{{\sigma }_{1}-{\sigma }_{3}}{{E}_{{\rm{K}}}}(1-{e}^{\frac{-{E}_{{\rm{K}}}}{{\eta }_{{\rm{K}}}}t})+\frac{{\sigma }_{1}-{\sigma }_{3}-{\sigma }_{S}}{{\eta }_{{\rm{N}}}}{(t-{t}_{{\rm{N}}})}^{n}\,{\sigma }_{1}-{\sigma }_{3}\ge {\sigma }_{S}$$

(2) When there is disturbance:

15 $$\varepsilon (t)=\frac{{\sigma }_{1}-{\sigma }_{3}}{{E}_{{\rm{M}}}(1-D)}+\frac{{\sigma }_{1}-{\sigma }_{3}}{{\eta }_{{\rm{M}}}(1-D)}t+\frac{{\sigma }_{1}-{\sigma }_{3}}{{E}_{{\rm{K}}}}(1-{e}^{\frac{-{E}_{{\rm{K}}}}{{\eta }_{{\rm{K}}}(1-D)}t})\,{\sigma }_{1}-{\sigma }_{3} < {\sigma }_{S}$$

16 $$\varepsilon (t)=\frac{{\sigma }_{1}-{\sigma }_{3}}{{E}_{{\rm{M}}}(1-D)}+\frac{{\sigma }_{1}-{\sigma }_{3}}{{\eta }_{{\rm{M}}}(1-D)}t+\frac{{\sigma }_{1}-{\sigma }_{3}}{{E}_{{\rm{K}}}}(1-{e}^{\frac{-{E}_{{\rm{K}}}}{{\eta }_{{\rm{K}}}(1-D)}t})+\frac{{\sigma }_{1}-{\sigma }_{3}-{\sigma }_{S}}{{\eta }_{{\rm{N}}}(1-D)}{(t-{t}_{{\rm{N}}})}^{n}\,{\sigma }_{1}-{\sigma }_{3}\ge {\sigma }_{S}$$

#### Creep damage model verification

**Figure 14:**
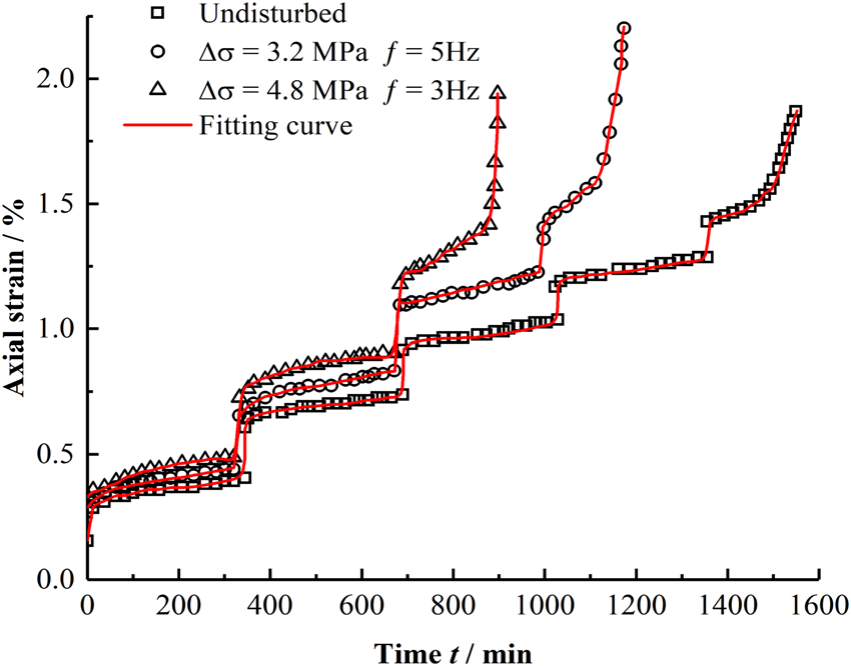
Comparison between test date and model fitting.

To verify the applicability of the perturbation creep damage model, according to the perturbation creep test results and creep curve analysis, this study chooses the model-based search (PS) improved nonlinear least squares method for model identification and parameter calculation^15^. The PS-based least square method takes the objective function of the conventional least square method as the objective function. The PS optimization method is used to optimize the parameters so that the objective function achieves the required accuracy and avoids the only way of the conventional least square method—solving linear equation groups. The solution mechanism is fundamentally changed, which avoids the difficulty of initial value selection and makes it convenient to select the rheological model. And the fitting curve has very high accuracy. In this study, a disturbance of 4.8 MPa and frequency of 3 Hz are obtained as creep data for a disturbance amplitude of 3.2 MPa and disturbance frequency of 5 Hz; these values are fitted and analyzed for a confining pressure of 3 MPa. The experimental values and fitting curves are shown in Fig. 14. To further reflect the influence of different disturbance amplitudes and frequencies on creep damage, the creep data under different disturbance conditions for a confining pressure of 3 MPa and axial pressure of 20 MPa is fitted and analyzed, and specific creep fitting is carried out. The parameter values are listed in Table 2.

**Table 2:** Model parameters of mudstone disturbance creep damage.

${\sigma }_{1}$/MPa	$\Delta \sigma$/MPa	$f$/Hz	${E}_{{\rm{M}}}$ /MPa	${\eta }_{{\rm{M}}}$ /GPa·min	${E}_{{\rm{K}}}$/MPa	${\eta }_{{\rm{K}}}$/GPa·min	${\sigma }_{{\rm{S}}}$/MPa	${\eta }_{{\rm{N}}}$/GPa·min	*t*_N_/min	*n*	*α* × 10^−4^
0–30	0	0	21.74	72.51	697.32	70.25	30	23.64	1472	1.37	—
0–25	1.6	3	23.05	76.53	585.14	56.76	25	28.17	1323	1.64	4.13
0–25	3.2	3	21.67	77.46	623.76	58.87	25	22.93	1188	2.11	5.28
0–20	4.8	3	21.25	79.52	668.35	65.23	20	27.91	883	2.87	6.33
0–25	3.2	1	22.30	70.28	593.42	58.65	25	25.61	1294	1.79	4.51
0–25	3.2	5	21.99	81.33	645.19	55.71	25	19.38	1089	1.52	5.77

## Conclusions

1. Rock creep undergoes transient deformation and decay, steady, and accelerated creep. Disturbance is an important factor affecting the creep properties of rocks, and different disturbance conditions have different effects at each creep stage.
2. Under the same disturbance condition, with the increase in axial pressure, the instantaneous deformation, creep decay time, and steady creep rate of mudstone vary exponentially. Under the same axial pressure, as the disturbance frequency and the disturbance amplitude increases, the instantaneous deformation, creep decay time, and steady creep rate increase linearly. Further, the influence of the disturbance amplitude on instantaneous deformation and steady creep rate is more significant.
3. The disturbance amplitude reduces the threshold stress of the rock entering the creep. The deformation of the rock accelerates creep and increases with the disturbance amplitude and frequency. The acceleration creep is not evident with the increase in the disturbance frequency. However, as the amplitude of the disturbance increases, the creep time decreases sharply.
4. A damage variable considering the disturbance amplitude and the disturbance frequency is introduced. A nonlinear damage creep model of rock based on Burger’s model is established. The required parameters are identified and calculated, and the rationality and applicability of the model are verified.

## Data Availability

The data used to support the findings of this study are available from the corresponding author upon request.

## References

[1] Heping XIE, Feng GAO, Yang JU (2015). Research and Development of Rock Mechanics in Deep Ground Engineering [J]. Chinese Journal of Rock Mechanics and Engineering.

[2] Šancer, J., Štrejbar, M. & Maleňáková, A. Effects of cyclic loading on the rheological properties of sandstones[J]. *Open Geosciences*, **3**, 10.2478/s13533-011-0020-8 (2011).

[3] Bagde MN, Petroš V (2005). Fatigue properties of intact sandstone samples subjected to dynamic uniaxial cyclical loading. Int. J. Rock Mech. Min..

[4] Bagde MN, Petroš V (2005). The effect of machine behaviour and mechanical properties of intact sandstoneunder static and dynamic uniaxial cyclic loading. Rock Mech. Rock Eng..

[5] Yanfa GAO (2008). A RHEOLOGICAL Test of sandstone with pertubation effect and its constitutive relationship study [J]. Chinese Journal of Rock Mechanics and Engineering.

[6] Bo WANG (2017). Axial load test study on the perturbation properties of rock rheology[J]. Journal of experimental mechanics.

[7] Xihai CUI (2007). Experimental study in rheological regularity and constitutive relationship of rock under disturbing loads [J]. Chinese Journal of Rock Mechanics and Engineering.

[8] Dazhao S (2011). Test study of the perturbation effect of coal measures rocks damage failure[J]. Journal of China University of Mining& Technology.

[9] Chengzhi PU (2017). Variable paramenters nonlinear creep damage model of rock with considerationg of aging, damage and deterioration[J]. Engineering mechanics.

[10] Qihu W (2016). A creep constitutive model of rock considering initial damage and creep damage[J]. Rock and Soil Mechanics.

[11] YANG Shengqi. Study on rheological mechanical properties of rock and its engineering applications [D]. Nan Jing:HoHaiUnviersiyty (2006).

[12] Kachanov M (1992). Effective elastic properties of cracked solids: critical review of some basic concepts[J]. Applied Mechanics Review.

[13] Weiya XU (2006). Study on creep damage constitutive relation of creenschist specimen[J]. Chinese Journal of Rock Mechanics and Engineering.

[14] Chen Luwang LI (2018). Further Development and Application of a Creep Damage Model for Water-Bearing Rocks[J]. Chinese Journal of Solid Mechanics.

[15] Junguang W, Bing L, Mi T (2014). Study of Creep Characteristics Produced by Nonlinear Damage of Oil Shale in Hydrous State[J]. Journal of experimental mechanics.

